# The effect of repeated cadmium oral exposure on the level of sex hormones, estrous cyclicity, and endometrium morphometry in female rats

**DOI:** 10.1007/s11356-018-2821-5

**Published:** 2018-07-31

**Authors:** Marzenna Nasiadek, Marian Danilewicz, Krystyna Sitarek, Ewa Świątkowska, Adam Daragó, Joanna Stragierowicz, Anna Kilanowicz

**Affiliations:** 1 Department of Toxicology, Faculty of Pharmacy, Medical University of Lodz, Muszynskiego 1, 90-151 Lodz, Poland; 2 Department of Pathology, Medical University of Lodz, Pomorska 251, 92-213 Lodz, Poland; 3 Department of Toxicology and Carcinogenesis, Nofer Institute of Occupational Medicine, sw. Teresy 8, 91-348 Lodz, Poland; 4 Research Institute Polish Mother’s Memorial Hospital, 93-338 Lodz, Poland

**Keywords:** Cadmium, Estradiol, Progesterone, Estrous cycle, Uterus, Ovaries, Rat

## Abstract

Cadmium (Cd) is regarded as a potential endocrine disruptor. However, the exact mechanism by which this metal may interfere with the reproductive system has not yet been elucidated. The present study aimed to investigate the effect of subacute Cd oral administration at daily doses of 0.09, 1.8, and 4.5 mgCd/kg b.w. and the impact of Cd on sex hormones (estradiol (E_2_) and progesterone (P)) in the plasma and uterus, as well as on estrous cyclicity and histopathological changes in uterine and ovary in female rats after terminating the exposure and after a prolonged observation period (3 months). Moreover, Cd bioaccumulation in the uterine and brain tissue of rats was analyzed. The study revealed that oral Cd exposure induced changes in the plasma levels of steroid hormones: decrease in E_2_ and increase in P after the highest dose of Cd. Probably, for the first time, it was evidenced that circulation sex hormone disturbances in Cd-exposed rats caused irregular estrous cycle, persisting for 3 months after exposure termination; no alterations in these hormone levels in uterine tissue were noted. Cd did not induce estradiol-like hyperplasia of endometrium, but resulted in endometrial edema irrespective of the dose, and caused damage of the ovaries after the highest dose. In summary, subacute oral exposure of female rats to Cd may lead to long-term disturbances in reproductive system.

## Introduction

Cadmium (Cd) is a toxic metal characterized by extensive persistence in the environment. In addition, modern civilization processes contribute to an increase of Cd in environmental circulation, leading to its rising occurrence in the food chains. In general population, exposure to Cd occurs mainly via food, water, and tobacco smoking (WHO 1992; EFSA 2012).

Studies have suggested endocrine modulative properties of Cd and therefore, it has been categorized into the group of environmental endocrine disruptors (EDs), which are defined as “exogenous substances or mixtures, that alter function(s) of endocrine system and consequently cause adverse health effects in an intact organism, or its progeny, or (sub)populations” (WHO/IPCS 2002). The effect of Cd on the male reproductive system in rats was first described in 1960 (Kar and Das 1960). Later studies confirmed that Cd chronic exposure does not only disturb the reproductive endocrine function but may also lead to the reduction in testicular mass, generative alterations in testes (Nolan and Shaikh 1986; Toman and Massányi 2002; Ciarrocca et al. 2013; de Angelis et al. 2017), and decreased spermatozoa mobility (Lukáč et al. 2003).

In turn, studies on female rats showed that Cd, metal with a very long half-life, accumulates in the female reproductive organs of the hypothalamic-pituitary-gonadal axis (Massányi et al. 1995, 1997, 2007; Varga et al. 1993; Nasiadek et al. 2005; Höfer et al. 2009; Jiménez-Ortega et al. 2011, 2012; Rzymski et al. 2016). The gonadotropins secreted by the anterior pituitary gland are required for synthesis of steroid hormones: estradiol (E_2_) and progesterone (P). Over the recent years, the endocrine disrupting effect of Cd has been extensively investigated but the outcomes of the studies are not explicit, which is indicated by both agonistic (Brama et al. 2007; Garcia-Morales et al. 1994; Stoica et al. 2000; Johnson et al. 2003; Höfer et al. 2009) and antagonistic (Höfer et al. 2009; Rider et al. 2009; Silva et al. 2006) effects of Cd.

Mechanisms, by which Cd affects the reproductive system, have not been fully elucidated yet. Estrogenic effect of Cd seems to be mediated by a direct interaction with the estradiol receptor (ER) (Johnson et al. 2003), but non-classical pathways could be also included (Ali et al. 2010). Moreover, Kluxen et al. (2012) proved that Cd could also modulate the expression of aryl hydrocarbon receptor (AhR) in the rat uterus via ER-mediated mechanism (cross-talk between ER and AhR pathways).

The review of in vitro and in vivo studies shows that Cd can both increase and inhibit the secretion of the major classes of steroid hormones: E_2_ and P and the effects are dependent not only on the dose and route of administration but also on the stages of estrous cycle (Massányi et al. 2000; Han et al. 2006; Zhang and Jia 2007; Zhang et al. 2008). Cd also induces significant changes in the structure of ovary (maturation of follicles, follicular atresia, degradation of corpus luteum, the damage of oocytes), as well as the uterus (a significant increase of the uterine epithelium height, interstitial edema, capillary modification) (Paksy et al. 1992; Massányi and Uhrín 1996, 1997; Massányi et al. 1997, 2005; Höfer et al. 2009; Ali et al. 2010; Wang et al. 2015).

The uterus, especially the thickness of the endometrial epithelium, is an established criterion for evaluation of the estrogenicity of xenobiotics. Moreover, the effects of changing the concentrations of estrogen and progesterone throughout the estrous cycle also have a characteristic impact on the endometrium (Diel 2002; Ali et al. 2010). Animal studies showed a significant increase in the epithelial thickness only after single intraperitoneal injections of CdCl_2_, but no changes in this parameter upon oral short- and long-term administration of Cd, which may suggest the significance of the route of administration (Höfer et al. 2009). Massányi and Uhrín (1997) demonstrated that the percentage of the glandular epithelium in the endometrium was significantly lower, while the amount of stroma was significantly higher in the group of rabbits receiving Cd intraperitoneally in comparison with the group receiving Cd orally. However, percentage content of surface epithelium was invariant regardless of administration route (Massányi and Uhrín 1997).

Although the effect of Cd on endometrium thickness in animals has been documented, there is hardly any data concerning humans. In a study conducted on a small group of 25 women, no correlation between Cd endometrial level and endometrium thickness was found in paper by Rzymski et al. (2014). However, it is worth emphasizing that a higher, two- to threefold concentration of Cd in the lesioned tissues (especially hyperplastic and cancerous) compared to normal endometrium was observed by the authors (Rzymski et al. 2014, 2016).

As non-smoking general population exposure to Cd mostly occurs orally, it is significant to investigate the effect of Cd on sex hormones levels, especially in the uterus. The aim of this study was to assess the effects of Cd, administered orally for 30 days (0.09, 1.8, 4.5 mgCd/kg b.w.) on the sex hormones (E_2_ and P) in female rats. These effects were evaluated in the prolonged observation (3 months) and determined in uterine tissues and plasma. Moreover, the study investigated the effects of Cd on estrous cyclicity and histopathological changes in uterine tissue and ovary, and accumulation of Cd in the uterus and brains was evaluated.

## Material and methods

### Animals and experimental design

The study was carried out in a population of regularly cycling female Wistar rats, aged 10 weeks, with an average initial body weight of 200 ± 12 g. Following a 2-week quarantine period, the estrous cycles were determined by a vaginal swab collected every morning (8:30–9:30 a.m.) and female rats with at least two regular 4-day cycles were used (total *n* = 120). Animals were housed (five per cage) under standard laboratory indoor conditions, with a 12-h light/dark cycle at room temperature (22 ± 1 °C) and relative humidity (50–60%) with free access to tap water and a diet low in phytoestrogen content (Ssniff R/M-H low phytoestrogen).

The model of the experiment is shown in Table 1. The Cd-exposed groups were administered CdCl_2_ (Sigma-Aldrich, St. Louis, MO, USA) by gavage, at three different daily doses: 0.09, 1.8, and 4.5 mgCd/kg b.w. (*n* = 10 in each group), which corresponded to 1/1000, 1/50, and 1/20 LD_50_, respectively. An average oral lethal dose (LD_50_) value for CdCl_2_ in rat was reported as 88 mgCd/kg b.w. (Lehman 1951). The positive control group (E_2_) was supplied with 17β-estradiol (0.03 mg/kg b.w.) (Sigma, St. Louis, MO, USA) dissolved in peanut oil. The applied dose of 17β-estradiol (E_2_) was equivalent to the average substance dose administered in women. All solutions were given daily, in a volume not exceeding 1 ml/100 g b.w./rat, by gavage for 7 days a week. Animals from control groups received by gavage water or peanut oil in the same volume, which was used in Cd-exposed groups.

**Table 1:** Experimental model

Experimental period	Group (*n* = 10 each)	Dose (mg/kg b.w.)
30-day exposure	Pure control	Water
	Cd	0.09
	Cd	1.8
	Cd	4.5
	Oil control	Peanut oil
	Positive control (E_2_)	0.03
30-day exposure and 90-day post-exposure period	Pure control	Water
	Cd	0.09
	Cd	1.8
	Cd	4.5
	Oil control	Peanut oil
	Positive control (E_2_)	0.03

The general behavior of the dams was observed daily. Just before administration, the females were weighed every day at the same time. Water intake, food consumption, and body weight were measured once a week. All females were sacrificed at the estrus stage by heart puncture under light carbon dioxide anesthesia. Subsequently, whole blood was drawn into Vacutainer tubes for metal analysis (S-Monovette, Sarstedt). During autopsy, the uterus, ovaries, and whole brain were dissected out. The first part of uteri and both ovaries were fixed in formaldehyde for histopathological examinations. For hormone analysis, the second part of uteri was washed in ice-cold physiological saline repetitively, weighed accurately, added with PBS (pH 7.2–7.4), and rapidly frozen with liquid nitrogen. Whole brain and the remaining part of uteri (ok. 0.1 g) were kept at −80 °C until Cd analysis. For Cd determination, whole blood (1 ml) was kept in acid-washed cryo-tubes at −80 °C, while the remaining part of blood was centrifuged at 3000×*g*/10 min in a refrigerated centrifuge at 4 °C to separate the plasma, and then stored at −80 °C until total cholesterol and hormone analyses.

### Method of estrous cycle examination

The estrous cycle was analyzed both before exposure and after two observation periods in all rats (after the 30-day exposure and then 3 months after the termination of exposure). To identify regular 4-day estrous cycles, vaginal smears were collected every morning for 12 days from all animals before exposure, and cytological analyses were performed by light microscopy (Fox and Laird 1970). Recognition of each phase was based on the proportion of three types of cells in the vaginal smear: leukocytes, epithelial cells, and cornified cells (Byers et al. 2012).

The following parameters were determined in each period of the experiment: length of the estrous cycle in its individual phases and the frequency of each of the four cycle phases (proestrus, estrus, metaestrus, and diestrus) in controls and Cd-exposed females. The period of time between the estrus phase determined by vaginal swab and the proestrus phase was taken as the length of the cycle. The percentage length of each individual cycle phase was determined based on the assumption that the 12-day observation period at any given period of the study represented 100%.

### Histopathological examinations

The uterus and ovaries were fixed in 10% formalin for 24 h and then embedded in paraffin blocks, sliced into 5-μm sections and stained with hematoxylin-eosin (H&E) for the histopathological evaluation. The sections were examined under the light microscope (Olympus BX-51; Olympus, Tokyo, Japan).

### Measuring endometrial thickness

The thickness of the endometrium was evaluated using the computer image analysis system consisting of a PC computer equipped with a Pentagram graphic tablet, Indeo Fast card (frame grabber, true-color, real-time), produced by Indeo (Taiwan), and color TV camera Panasonic (Japan) coupled with Carl Zeiss microscope (Germany). This system was programmed (MultiScan 18.03 software, produced by Computer Scanning Systems, Poland) to calculate the distance (semiautomatic function).

In each case, 7–10 measurements were performed in high-power monitor fields and then the mean endometrium thickness was calculated.

### Biochemical analysis

The plasma estradiol (E_2_) and progesterone (P) levels were determined by electrochemiluminescence using a Roche Diagnostic kit on a Cobas 2601 analyzer (LOD values were as follows: estradiol, 5 pg/ml; progesterone, 0.03 ng/ml). The values reported are the sum of estradiol and estrone because chromatographic purification of the samples was not performed.

The levels of estradiol and progesterone in the rat tissue were determined using ELISA kit, according to the manufacturer’s instructions, respectively: rat (E_2_) ELISA kit—catalog no. 201-11-0175, SRB (China), progesterone (P) ELISA kit—catalog no. CSB-E07282r Cusabio Biotech Co., Ltd., (Japan). The sensitivities of E_2_ and P kits are 3.112 pg/ml and 0.25 ng/ml, respectively. The samples of uterus for hormone analysis were homogenized well to produce 10% homogenates in PBS and stored overnight at 20 °C. For assay of estradiol, the samples were centrifuged for 20 min at speed of 2000–3000 rpm, and in the supernatant, the analysis was conducted immediately.

For assay of progesterone, after two freeze**-**thaw cycles were performed to break the cell membranes, the homogenate was centrifuged for 5 min at 5000×*g*, 2–8 °C. The assay in the supernatant was carried out immediately.

Total serum cholesterol (CHOL) (precursor of reproductive hormones) was analyzed using the Vitros 5.1 FS CHOL Slides and the Vitros Chemistry Products Calibrator Kit 2 on the Vitros Chemistry System. The Vitros CHOL Slide is a multilayered analytical element coated on a polyester support. The enzymatic method was similar to that proposed by Allain et al. (1974).

### Cd analysis

Cd concentration was analyzed in the whole blood, uterus, and whole brain, using a GFAAS (Hitachi Z-8270) with Zeeman-type background correction, autosampler, and pyrocoated tube. The digestion of samples was performed with a MarsXpress microwave digestion system (CEM, USA). For the determination of Cd concentrations, duplicate samples were prepared. The analytical quality control of the whole blood and tissue samples (reference samples: Seronorm Whole Blood L-1, Sero, Norway, and Bovine Liver SRM 1577b, National Institute of Standards and Technology, USA) was within the range of reference values. The detection limit for Cd was 0.2 μg/L or 0.2 ng/g wet tissue. Cd concentrations in the whole blood, whole brain, and in uterine tissue were expressed as micrograms per liter or micrograms per gram wet tissue.

### Statistical analysis

All data were expressed as mean ± standard deviation. We used the Kruskal-Wallis one-way analysis of variance followed by the pairwise comparison of selected means with the Mann-Whitney *U*-test. Significance was set at a value of *p* ≤ 0.05. Statistically analysis was performed using Statistica v. 12.5 (StatSoft, Krakow, Poland).

In the estrous cycle, the statistical analysis comprised of the following: (1) the mean length of estrous cycle in controls and Cd-exposed females and (2) the frequency of each of 4 cycle phases.

The one-way analysis of variance following the Dunnet’s test was used in the case of variance homogeneity, and the Kruskal-Wallis analysis of variance was followed by the non-parametric test in the case of heterogeneity. Frequency data were analyzed with the Fisher’s exact probability test.

## Results

### Clinical observation

Neither during Cd administration nor in a 3-month observation period, changes in the integral toxicity indicators, manifested by alterations in the body weight and chosen organ weight (Table 2), water intake, and food consumption (data not presented) were observed. As shown in the table, there were no statistically significant differences in both the final body weight and chosen organ weight (relative and absolute) between females receiving Cd doses in the range of 0.09–4.5 mgCd/kg b.w. and the pure control groups. In the positive control group, although the uterine weight did not statistically increase after 30 days of 17β-estradiol administration, the tendency to increase the weight persisted for 3 months after the end of exposure in comparison to oil control (Table 2).

**Table 2:** Body weight, brain, and uterine weights of rats after 30-day oral exposure to CdCl_2_ or 17β-estradiol (E_2_) and after 90-day post-exposure period and in controls of rats

Treatment	Dose (mg/kg b.w.)	Body weight		Body weight change		Brain		Uterus	
		At the beginning (g)	At the end (g)	Weight (g)	% of initial body weight	Weight (g)	% of final body	Weight (g)	% of final body
30-day exposure									
Pure control	0	193.4 ± 7.3	203.0 ± 9.7	9.6 ± 4.3	4.8 ± 2.1	1.68 ± 0.04	0.82 ± 0.23	0.60 ± 0.11	0.29 ± 0.06
Cd	0.09	212.1 ± 5.4	225.6 ± 9.4	13.5 ± 8.6	6.1 ± 4.0	1.72 ± 0.14	0.76 ± 0.25	0.72 ± 0.07	0.32 ± 0.03
Cd	1.8	214.8 ± 10.2	222.7 ± 10.9	7.9 ± 6.0	3.5 ± 2.8	1.74 ± 0.06	0.78 ± 0.16	0.69 ± 0.12	0.31 ± 0.04
Cd	4.5	216.8 ± 7.6	228.3 ± 10.2	11.5 ± 8.1	5.1 ± 3.9	1.64 ± 0.07	0.72 ± 0.14	0.69 ± 0.15	0.30 ± 0.12
Oil control	0	195.8 ± 6.7	213.3 ± 12.1	17.5 ± 8.2	8.3 ± 4.1	1.69 ± 0.07	0.78 ± 0.15	0.63 ± 0.10	0.30 ± 0.04
Positive control (E_2_)	0.03	223.6 ± 10.9	231.6 ± 12.2	8.0 ± 4.0	3.5 ± 1.8	1.74 ± 0.12	0.76 ± 0.16	0.79 ± 0.15	0.34 ± 0.06
90-day post-exposure period									
Pure control	0	184.5 ± 6.0	251.0 ± 22.9	66.5 ± 18.1	36.2 ± 11.5	1.74 ± 0.06	0.68 ± 0.12	0.86 ± 0.09	0.34 ± 0.14
Cd	0.09	203.0 ± 3.3	260.4 ± 14.8	57.5 ± 11.8	22.0 ± 6.1	1.83 ± 0.08	0.71 ± 0.10	0.81 ± 0.09	0.31 ± 0.04
Cd	1.8	200.8 ± 3.7	252.4 ± 15.6	51.6 ± 14.3	20.5 ± 8.1	1.88 ± 0.14	0.74 ± 0.41	0.95 ± 0.11	0.37 ± 0.05
Cd	4.5	190.6 ± 7.7	251.3 ± 20.9	60.6 ± 24.7	24.1 ± 13.5	1.78 ± 0.02	0.73 ± 0.14	0.95 ± 0.11	0.38 ± 0.08
Oil control	0	190.0 ± 5.3	247.8 ± 18.9	57.8 ± 16.4	23.3 ± 8.5	1.82 ± 0.02	0.73 ± 0.14	0.80 ± 0.14	0.29 ± 0.14
Positive control (E_2_)	0.03	196.0 ± 4.3	240.9 ± 11.9	44.9 ± 9.8	18.6 ± 4.9	1.79 ± 0.06	0.74 ± 0.03	0.99 ± 0.13	0.41 ± 0.15

All values expressed as means ± SD (*n* = 10 rats per group)

### Plasma level of steroid hormones

**Fig. 1:**
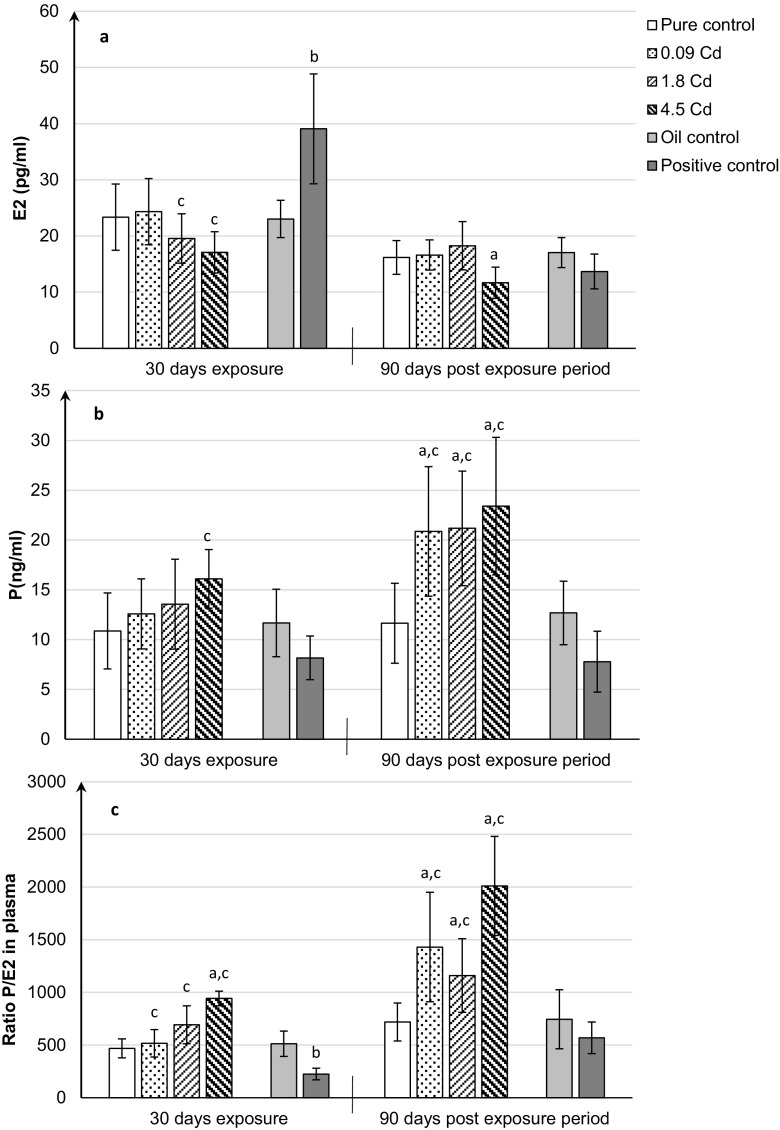
Estradiol (E_2_) (**a**), progesterone (P) (**b**) concentrations, and ratio P/E_2_ (**c**) in the plasma after 30-day oral exposure to CdCl_2_ or 17β-estradiol (E_2_) and after 90-day post-exposure period and in controls of rats. All values expressed as means ± SD (*n* = 10 animals per dose group). Mean values with different superscript letters in the same row are significantly different at *p* ≤ 0.05 (a—vs. pure control group, b—vs. oil control, c—vs. positive control)

Figure 1a, b presents plasma concentrations of sex hormones (E_2_ and P) in female rats of the study and control groups. The tendency to decrease plasma E_2_ concentration was observed in female rats receiving the highest Cd dose (4.5 mgCd/kg), but this effect was not significant (Fig. 1a). After 90 days following the exposure termination, significant lower plasma concentrations of E_2_ were still maintained in the Cd group (4.5 mgCd/kg) compared to the pure control (Fig. 1a).

An upward trend in the plasma progesterone concentration (Fig. 1b) in females receiving Cd at all applied doses (0.09–4.5 mgCd/kg) was noted after 30 days of administration, but the increase was statistically insignificant. It is worth noting that after 90 days a significant increase in plasma progesterone was also observed in all Cd-exposed groups of female rats, regardless of the administered Cd doses, compared to the pure control group (Fig. 1b).

To illustrate the disturbed metabolism of the analyzed hormones due to Cd effect, the ratio of progesterone-estradiol levels (P/E_2_) was also calculated (Fig. 1c). The P/E_2_ ratio was significantly higher in females after termination of a 30-day exposure to the highest doses (4.5 mgCd/kg) and also 90 days after Cd administration in all groups (Fig. 1c). About a two- to threefold increase was observed.

Unlike Cd, 17β-estradiol (positive control) induced a typical effect for this compound, i.e., a statistical significant increase in plasma E_2_ level and an insignificant decrease in P level, as compared to the oil control (Fig. 1a, b). Confirmation of this dependence was a substantial decrease of the ratio of progesterone-estradiol levels (P/E_2_) after termination of a 30-day administration (Fig. 1c).

**Fig. 2:**
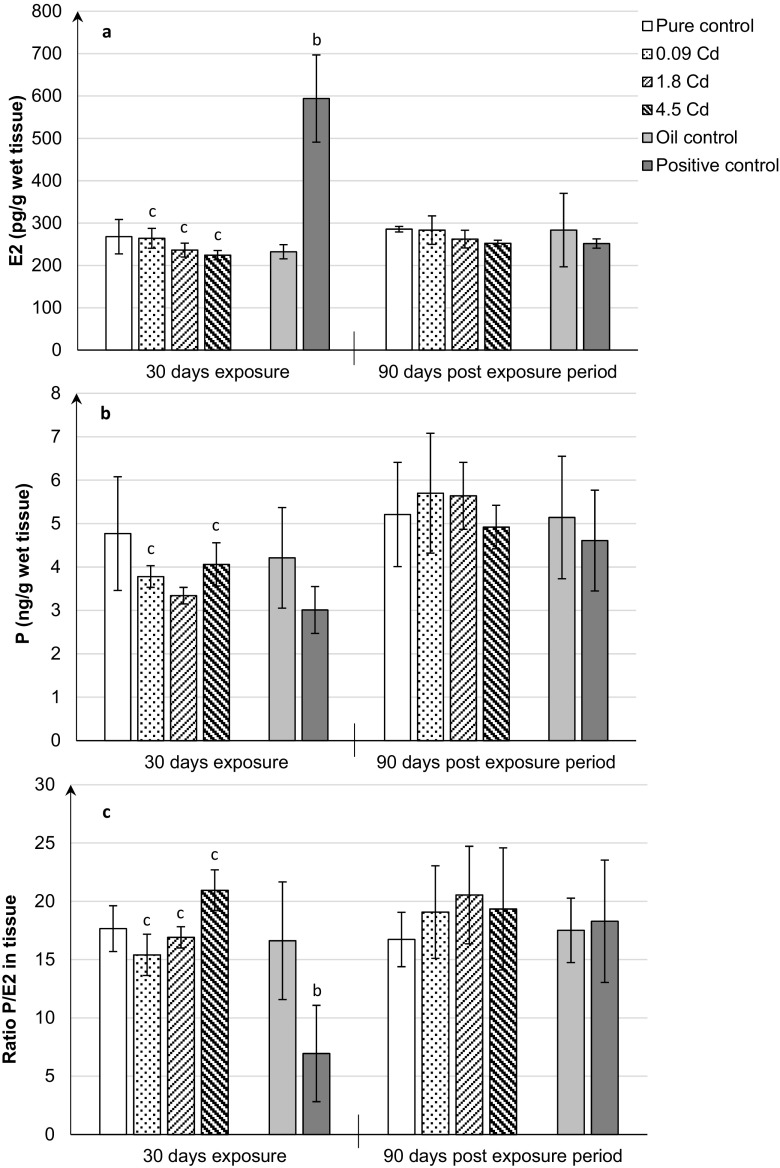
Estradiol (E_2_) (**a**), progesterone (P) (**b**) concentrations, and P/E_2_ ratio (**c**) in the uterine after 30-day oral exposure to CdCl_2_ or 17β-estradiol (E_2_) and after 90-day post-exposure period and in controls of rats. All values expressed as means ± SD (*n* = 10 animals per dose group). Mean values with different superscript letters in the same row are significantly different at *p* ≤ 0.05 (a—vs. pure control group, b—vs. oil control, c—vs. positive control)

Concentrations of steroid hormones in the uterus tissue are given in Fig. 2a, b. Similarly to plasma, the tissue ratio of progesterone-estradiol levels (P/E_2_) was calculated (Fig. 2c). As shown in Fig. 2 a, b, no significant changes in the concentration of both hormones (P and E_2_) in the uterus tissue after Cd administration to females at doses of 0.09–4.5 mgCd/kg were found after termination of a 30-day exposure and 90 days of post-exposure observation. The increased E_2_ levels (~ 2.5-fold) in the uterus tissue of rats (Fig. 2a) with the co-existing significantly decreased P/E_2_ ratio were found only in the positive control group (Fig. 2c).

**Fig. 3:**
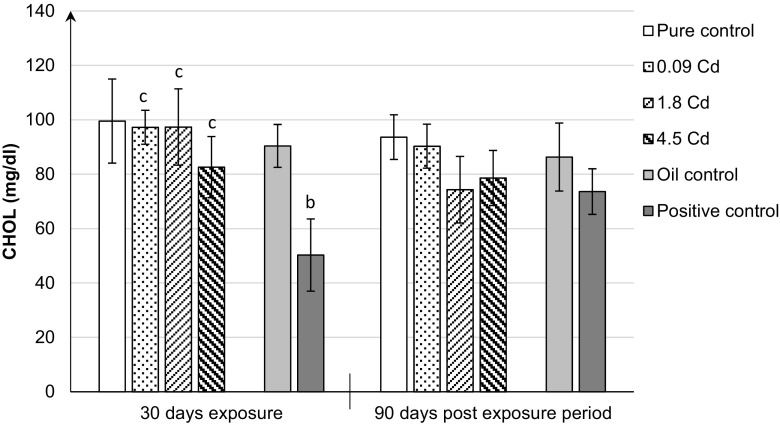
Total cholesterol concentrations (CHOL) in the plasma of rats after 30-day oral exposure to CdCl_2_ or 17β-estradiol (E_2_) and after 90-day post-exposure period and in controls of rats. All values expressed as means ± SD (*n* = 10 animals per dose group). Mean values with different superscript letters in the same row are significantly different at *p* ≤ 0.05 (a—vs. pure control group, b—vs. oil control, c—vs. positive control)

Figure 3 presents the levels of total cholesterol (CHOL) in rat females in all groups under study. As shown, there were no significant effects of Cd on CHOL levels compared to controls. Only, the administration of 17β-estradiol generated a significant decrease in the concentration of total CHOL, up to 50%, in the female plasma compared to the control oil group (Fig. 3).

### Estrous cycle

The estrous cycle was analyzed after a 30-day administration of the three different daily Cd doses of 0.09, 1.8, and 4.5 mgCd/kg b.w. and 3 months after the exposure termination. Prior to the exposure, an average length of the sexual cycle in rats ranged from 3.93 to 4.29 days. Compared to pure controls, Cd doses of 0.09 and 1.8 mgCd/kg b.w. did not exert a significant impact on the length of the sexual cycle, while in females exposed to the highest Cd dose (4.5 mgCd/kg b.w.), the cycle length was significantly prolonged up to 5.75 days. After a 30-day administration of 17β-estradiol (0.03 mg/kg), no significant changes in the cycle length of the females were observed as compared to the oil control group (Table 3).

**Table 3:** The effect on estrous phase in female rats after 30-day oral exposure of CdCl_2_ or 17β-estradiol (E_2_) and after 90-day post-exposure period and in controls of rats

Group	Period	Days in diestrus phase	Days in proestrus phase	Days in estrus phase	Days in metaestrus phase	Cycle length (days)
Pure control	30-day exposure	4.29 ± 0.78	1.50 ± 0.38	2.75 ± 0.65	2.19 ± 0.26	4.14 ± 0.38
	90-day post-exposure period	4.30 ± 0.70	1.75 ± 0.27	2.69 ± 0.53	3.25 ± 0.38	3.93 ± 0.24
Cd (0.09 mg/kg)	30-day exposure	4.69 ± 0.75	1.56 ± 0.42	2.50 ± 0.53	2.31 ± 0.46	4.16 ± 0.40
	90-day post-exposure period	4.50 ± 0.96	1.63 ± 0.23	2.63 ± 0.44	3.25 ± 0.60	4.00 ± 0.23
Cd (1.8 mg/kg)	30-day exposure	3.72 ± 0.71	1.72 ± 0.44	3.17 ± 0.61	2.39 ± 0.65	4.19 ± 0.27
	90-day post- exposure period	5.06 ± 0.85	1.61 ± 0.22	2.89 ± 0.80	2.44 ± 0.81	4.11 ± 0.22
Cd (4.5 mg/kg)	30-day exposure	5.61 ± 0.42^a^	1.29 ± 0.59	2.19 ± 0.96	2.13 ± 1.16	5.75 ± 0.64^a^
	90-day post- exposure period	5.25 ± 0.53^a^	1.56 ± 0.42	2.40 ± 0.46	2.69 ± 0.53	4.97 ± 0.20
Oil control	30-day exposure	4.35 ± 0.49	1.65 ± 0.27	2.81 ± 0.84	2.19 ± 0.70	4.72 ± 0.62
	90-day post- exposure period	4.48 ± 0.83	1.94 ± 0.68	2.88 ± 0.35	2.51 ± 0.53	4.06 ± 0.18
Positive control E_2_ (0.03 mg/kg)	30-day exposure	4.06 ± 1.26	2.00 ± 0.87	2.56 ± 0.73	2.39 ± 0.74	4.28 ± 0.57
	90-day post-exposure period	4.50 ± 0.75	1.94 ± 0.46	2.89 ± 0.65	2.67 ± 0.71	4.03 ± 0.40

All values expressed as mean ± SD (*n* = 10 rats per group)

^a^Statistical results indicate significance compared with the corresponding control group (*p* ≤ 0.05)

**Fig. 4:**
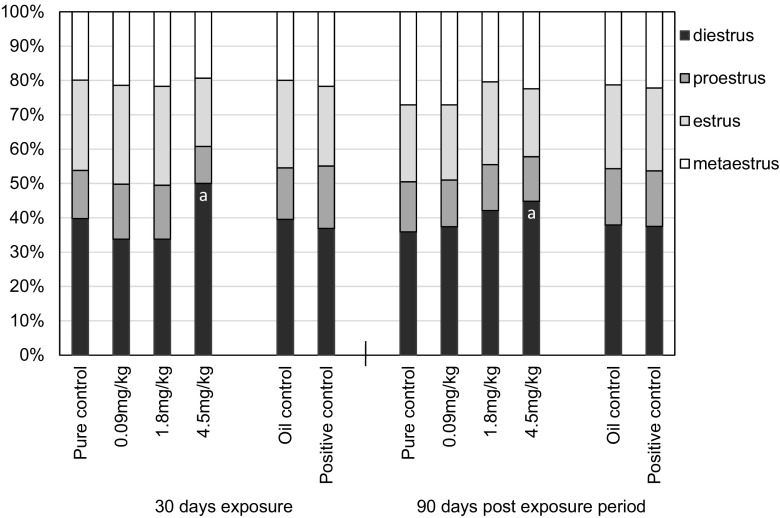
The frequency of each of the four cycle phases in female rats after 30-day oral exposure to CdCl_2_ or 17β-estradiol (E_2_) and after 90-day post-exposure period and in controls of rats. All values expressed as mean ± SD (*n* = 10 rats per group). a - Statistical results indicate significant difference compared with the corresponding control group (*p* ≤ 0.05)

Cd administered at doses of 4.5 mg/kg b.w. also caused an irregular pattern with cycles of extended duration and changed frequency of cycle phases (Fig. 4). After 30 days of exposure to 4.5 mgCd/kg, the diestrus phase was found to be significantly longer, compared to the pure control. A similar effect was also found after prolonged observation (3 months) (Fig. 4).

### Histopathological picture of the uterine specimens

**Fig. 5:**
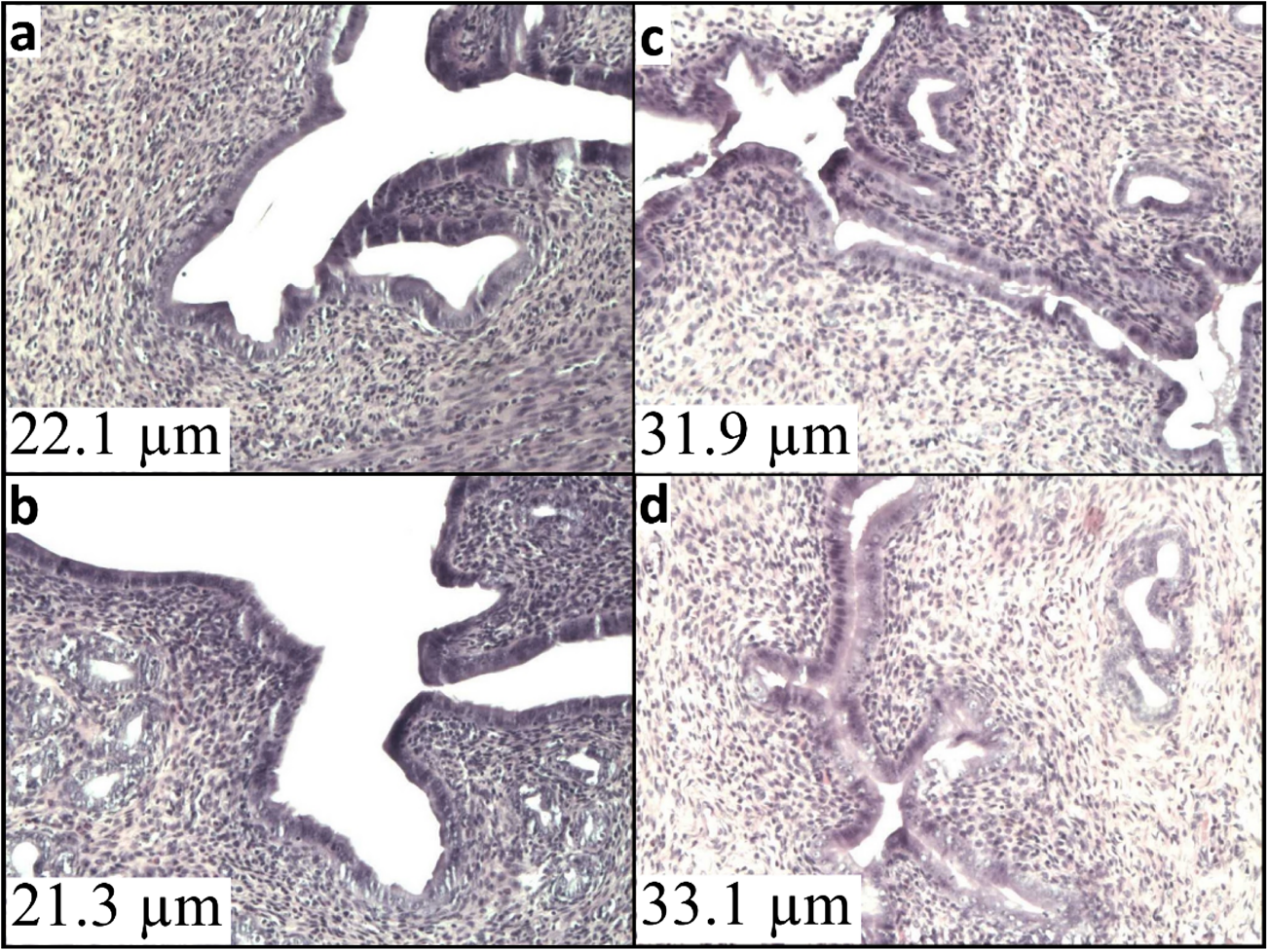
Effect of CdCl_2_ on uterine histopathology in the experimental groups. Photomicrographs of uterine sections stained with hematoxylin and eosin (magnification ×100). (**a**) Sections from the uteri of pure control rats. (**b**) Sections from the uteri of Cd group (0.09 mgCd/kg) exhibit an increase in endometrial thickness. (**c**) Sections from the uteri of oil control rats. (**d**) Sections from the uteri of 17β-estradiol group (0.03 mgE_2_/kg) exhibit an increase in endometrial thickness

Uterine sections obtained from the control group exhibited a normal endometrium structure of high columnar epithelial cells with numerous cytoplasmic vacuoles containing drops of mucus, while the underlying spindled stroma showed active glands (Fig. 5).

### Endometrial thickness

**Fig. 6:**
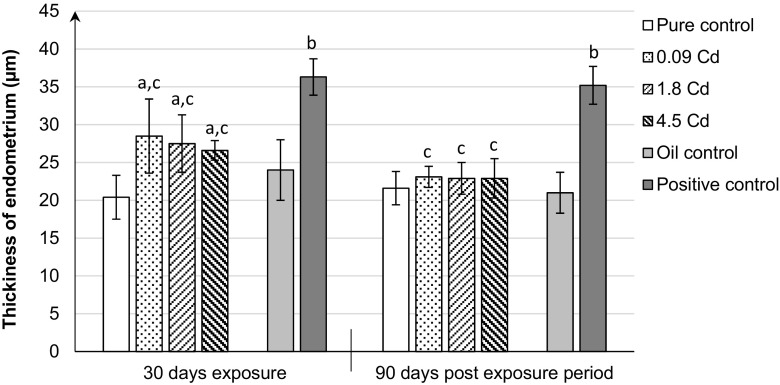
Mean of endometrium thickness after 30-day oral exposure to CdCl_2_ or 17β-estradiol (E_2_) and after 90-day post-exposure period and in controls of rats. All values expressed as means ± SD (*n* = 10 animals per dose group). Mean values with different superscript letters in the same row are significantly different at *p* ≤ 0.05 (a—vs. pure control group, b—vs. oil control, c—vs. positive control)

Morphometric analysis during the estrus stage exhibited that Cd administered orally (0.09, 1.8, 4.5 mg/kg b.w.) showed a significantly increased endometrium thickness, 1.39-, 1.34-, and 1.30-fold, respectively, when compared to the pure control group (Fig. 6). Changes resulting from Cd administration suggest endometrial edema, which disappears with time. In contrast, in the group of females treated with 17β-E_2_, a different type of endometrium changes with increased glands and underlying spindled stroma in the uterus was observed. These changes might suggest endometrial hyperplasia. Additionally, the increase in endometrial thickness (1.51-fold) was detected in comparison to the oil and pure control (Fig. 6), which persisted for 3 months following the termination of the exposure to 17β-estradiol.

### Ovary histology

**Fig. 7:**
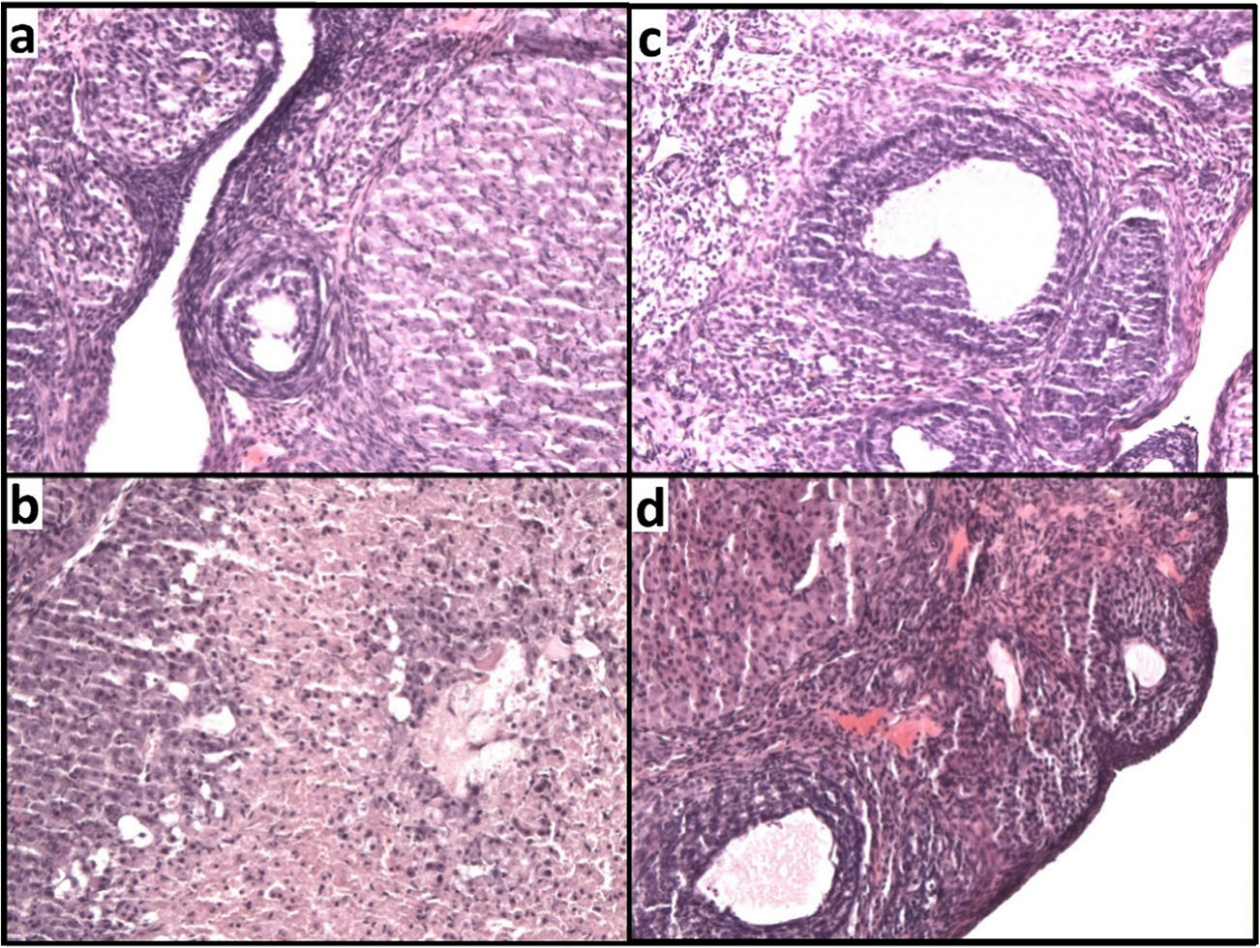
Effect of CdCl_2_ on ovary histopathology in the experimental groups. Photomicrographs of ovary sections stained with hematoxylin and eosin (magnification ×100). (**a**) Sections from the ovary of pure control rats. (**b**) Sections from the ovary of Cd group (4.5 mgCd/kg) exhibit a degeneration of corpora lutea. (**c**) Sections from the ovary of Cd group (4.5 mgCd/kg) exhibit a degeneration of granulosa cells and damaged oocytes. (**d**) Sections from the ovary of 17β-estradiol group (0.03 mgE_2_/kg) with visible corpus luteum and follicles

The control group demonstrated the normal basic structure of rats’ ovary usually containing corpus luteum and all developmental stages of follicles (Fig. 7a). The Cd-exposed group (4.5 mgCd/kg b.w.) showed evident ovary damage (Fig. 7b). It was found that the areas of degeneration of corpora luteum were seen and the oocytes were damaged and less numerous in Cd ovary tissues as compared to controls. Some moderate signs of degeneration of granulosa cells were also present (Fig. 7c). In positive control, no degenerative changes in ovaries were reported (Fig. 7d).

### Cd concentration in the blood, brain, and uterus

The study revealed the increased Cd concentrations in the whole blood, whole brain, and uterus of the female rats (Table 4). A subacute oral Cd administration at doses of 0.09–4.5 mg/kg b.w. resulted in a significant dose-dependent increase in the blood Cd concentration (Table 4).

**Table 4:** Cadmium concentrations in blood, brain and uterus after 30 days of oral exposure to CdCl_2_ or 17β-estradiol (E_2_) and 90-day post-exposure period and in controls of rats

Treatment	Dose (mg/kg b.w.)	Blood μgCd/l	Brain μgCd/g wet tissue	Uterus μgCd/g wet tissue
30-day exposure				
Pure control	0	0.17 ± 0.09	0.001 ± 0.001	0.013 ± 0.007
Cd	0.09	0.53 ± 0.09^a,c,d^	0.003 ± 0.001	0.016 ± 0.009
Cd	1.8	19.71 ± 3.39^a,b,d^	0.011 ± 0.003^a,b,d^	0.125 ± 0.012^a,b,d^
Cd	4.5	62.05 ± 13.94^a,b,c^	0.032 ± 0.06^a,b,c^	0.450 ± 0.030^a,b,c^
Oil control	0	0.19 ± 0.04	0.002 ± 0.001	0.010 ± 0.006
Positive control (E_2_)	0.03	0.18 ± 0.08	0.002 ± 0.001	0.012 ± 0.006
90-day post-exposure period				
Pure control	0	0.38 ± 0.05	0.002 ± 0.001	0.008 ± 0.003
Cd	0.09	0.54 ± 0.04	0.002 ± 0.001	0.014 ± 0.002
Cd	1.8	1.52 ± 0.33^a,b,d^	0.011 ± 0.001^a,b,d^	0.078 ± 0.034^a,b,d^
Cd	4.5	2.25 ± 0.36^a,b,c^	0.027 ± 0.004^a,b,c^	0.354 ± 0.031^a,b,c^
Oil control	0	0.33 ± 0.06	0.002 ± 0.001	0.011 ± 0.005
Positive control (E_2_)	0.03	0.39 ± 0.09	0.002 ± 0.001	0.013 ± 0.006

All values expressed as means ± SD (*n* = 10 rats per group). Mean values with different lowercase letters in the same row are significantly different at *p* ≤ 0.05 (a—vs. pure control, b—vs. 0.09 Cd group, c—vs.1.8 Cd group, d—vs. 4.5 Cd group)

Similar to the whole blood, we observed an increase in the whole brain Cd levels in a dose-dependent manner in Cd rats, compared to controls (Table 4). After a 30-day Cd administration at a dose of 0.09 mgCd/kg b.w., the increase in the whole brain Cd levels was insignificant, compared to pure control, while the administration of the higher doses (1.8 and 4.5 mgCd/kg b.w.) significantly enhanced the Cd concentration in the whole brain, and the increase was dose-dependent (five- and tenfold, respectively), as compared to the pure control group. The elevated Cd concentration in brain tissues persisted at the same level for successive 3 months after the exposure termination (Table 4). Moreover, a significant correlation between the Cd concentration in the whole blood and in brain tissues was observed in the examined females after both 30 days of exposure (*R*^2^ = 0.80) and 3 months following the termination of exposure (*R*^2^ = 0.87).

As in the brain, a significant increase in the Cd concentration in uterine tissues was found to be dose-dependent (Table 4). After 30 days of administration, the mean Cd concentration increase in uterine tissues depended on the dose, ~ 10× for 1.8 mgCd/kg and ~ 35× for 4.5 mgCd/kg, compared to the control group. There was a significant correlation between the Cd concentration in the blood and in the uterine tissues just after 30 days of Cd administration (*R*^2^ = 0.94), as well as 3 months after the exposure termination (*R*^2^ = 0.85). It should also be noted that Cd concentrations in uterine tissues were 5 to 14 times higher at 1.8 and 4.5 mgCd/kg, respectively, than in the brain. However, in the females receiving the two highest doses, Cd concentrations in the brain and uterus were found to be slightly decreased 3 months after the exposure termination (observation period), compared to the levels noted after 30 days of administration, while a dramatic reduction was observed in the blood (Table 4).

## Discussion

Recent studies have suggested that certain toxic metals such as Cd can function as endocrine disruptors by mimicking the action of estrogens (Garcia-Morales et al. 1994; Johnson et al. 2003; Stoica et al. 2000; Byrne et al. 2009). Literature data point out that Cd activates both genomic (Brama et al. 2007; Garcia-Morales et al. 1994; Stoica et al. 2000; Johnson et al. 2003; Höfer et al. 2009) and non-genomic estrogen signaling (Höfer et al. 2009; Rider et al. 2009; Silva et al. 2006). Experimental animal studies of Cd estrogenicity have yielded differentiated and ambiguous results concerning Cd effects on the hormonal system, manifested by the changed levels of sex hormones (E_2_ and P). It seems that the exposure route plays here a significant role. For example, after a single, subcutaneous Cd administration at doses within the range of 2.5–7.5 mgCd/kg, a reduced plasma progesterone level was observed (Zhang and Jia 2007), while after a single intraperitoneal administration of similar doses (1.6–8.4 mgCd/kg), an opposite effect was revealed (Obianime et al. 2011). Despite the fact that the same route of administration was used, various authors have reported conflicting results after repeated Cd oral administration at doses of 10–23 mgCd/kg (guinea pigs and rats). For example, Han et al. (2006) and Dobranić et al. (2002) showed the decreased blood E_2_ and P concentrations, while Monsefi and Fereydouni (2013) revealed the increased P concentration and the decreased E_2_ concentration.

In our study, we applied the experimental model of the oral exposure to a wide range of doses (0.09–4.5 mgCd/kg b.w.), a well-known main route for the general population exposure. Moreover, taking into account the long t_1/2_ of Cd in the organism, we applied the experimental model with a prolonged observation period after the exposure (3 months). The results show that Cd after 30 days of oral administration affects circulating sex hormones, and what is significant, the changes we observed persisted even up to 3 months after terminating the exposure, especially after the highest administered dose. However, the most significant Cd effects on progesterone were observed during a 3-month observation period after the Cd exposure, and interestingly, these effects did not depend on the dose. It appears that the plasma P/E_2_ ratio, calculated in our study, largely reflected changes in progesterone homeostasis, indicating its significantly higher value in all Cd-exposed female rats, regardless of doses, compared to controls. It is important to note that this ratio also revealed significant differences between groups directly after a 30-day Cd administration, especially after higher doses (4.5 mgCd/kg), when only an upward trend in plasma progesterone levels was observed.

Disorders of individual hormone secretion, including female sex hormones and disturb hormonal balance in organisms, can lead to various adverse health implications. Progesterone is a steroid hormone produced by the corpus luteum, placenta, and ovarian follicle. The uterus, ovaries, the mammary gland, and the brain are target organs for progesterone. Although our studies did not reveal significant changes in sex hormone levels in uterine tissues after 1 month of the Cd exposure, hormonal disorders at the organ level after a longer, chronic exposure cannot be excluded. Furthermore, progesterone homeostasis determines, among others, a regular estrous cycle in female rats. Our studies revealed the irregular estrous cycle following prolonged diestrus phases in females after a 30-day Cd exposure, only after administration of high Cd doses (4.5 mgCd/kg). It is important to note that those effects persisted for up to 3 months after the exposure termination. Bearing in mind the subsidence of E_2_ stimulation symptoms in the diestrus phase, followed by the physiological increase in P level, it is quite likely that the prolonged diestrus phase, observed in our study, could result from the enhanced plasma progesterone levels induced by the Cd exposure. Similar results (prolonged diestrus phase) after oral administration of a tenfold higher dose (40 mgCd/kg) for 6 weeks, as well as after inhalation exposure to CdO at a concentration of 1 mgCd/m^3^ for 20 weeks were observed earlier by Barański and Sitarek (1987). However, unlike in our studies, those authors did not check for how long those effects could last and whether they could persist for the period of successive 3 months after exposure termination.

The actual mechanisms of Cd effects on the female steroid production have still remained unclarified. The interruption in the steroidogenic pathway by Cd toxic action may be explained in a few different ways. The E_2_ level decline and the P level increase may result from the impairment of steroidogenic enzymatic activities by Cd as indicated by a number of studies (Pillai et al. 2012; Zhang and Jia 2007). Conversion of cholesterol to pregnenolone is supposed to be the cause of abnormalities in the metabolism of sex hormones (Smida et al. 2004). Our studies did not evidence a significant impact of the administered Cd on total cholesterol level, compared to other in vivo studies, which suggests that Cd may decrease the level of cholesterol in experimental animals (Skoczyńska and Smolik 1994; Akahori et al. 1994). However, the results of in vivo studies in recent years have shown a significant increase in serum total cholesterol after Cd administration, which indicates its ambiguous effect on this parameter (Samarghandian et al. 2015).

In our opinion, the disturbance in circulating sex hormones may also be affected by the accumulation of Cd in the brain. Our results indicated that the subacute Cd overload could lead to brain accumulation in female rats in a dose-dependent manner. Although we did not study the accumulation in different brain structures, the results of Gonçalves and co-workers (Gonçalves et al. 2012) showed that a long-term intoxication with Cd-containing diet leads to this metal accumulation in different brain structures in rats, and the concentrations in individual structures do not differ significantly. It cannot be excluded that the changed sex hormone levels result from Cd effects on the hypothalamus-pituitary-ovarian axis. This axis is a key regulator of hormone secretion (Lafuente et al. 2004, Lafuente 2013). It is worth emphasizing that the brain Cd exhibited a positive correlation with the blood Cd and uterus Cd levels in our model.

The uterus, especially the thickness of the endometrial epithelium, is an established criterion for evaluation of the estrogenicity of xenobiotics (Diel 2002; Ali et al. 2010). It is known that the endometrium changes throughout the menstrual cycle in response to hormones. The effects of Cd on the uterus differ in previous studies and apparently depend on the route and the time of exposure (Massányi and Uhrín 1997; Zhang et al. 2007; Höfer et al. 2009; Liu et al. 2010; Ali et al. 2010). Whereas after the intraperitoneal and subcutaneous administration, a significant increase in the luminal epithelial height was observed, the intracoronary effect after 3-day oral Cd exposure was reversed. Our study also showed a marked increase in the endometrial thickness during estrus after 30-day exposure to Cd, however, irrespective of dose. After 3 months of observation, following termination of the exposure to Cd, the thickness of endometrium was similar to control values. Light microscopic morphological study in the Cd-treated group suggests that endometrial thickness results from its edema. Massányi and Uhrín (1997) suggested that edematization of the uterus is caused by the blood vessel dilatation and subsequent diapedesis. It cannot be excluded that lack of changes in the endometrium in Cd groups after 3 months of observation might result from a persistent increased level of progesterone in plasma, which physiologically prevents increased thickness of the endometrium. On the other hand, in 17β-estradiol group, hyperplasia of the endometrium (increased glands and underlying spindled stroma) persisting for 3 months following the termination of the exposure was observed.

In the study of human endometrium, significantly higher concentration of Cd was found in tissues with recognized histological changes (endometrial hyperplasia, polyposis, atrophy, or cancer), in comparison with normal endometrium (Rzymski et al. 2014, 2016). However, no correlation between metal content and endometrial thickness was observed (Rzymski et al. 2014).

In our study, histological changes in the structure of ovaries, observed after oral administration of Cd, especially following the highest dose, were similar to the changes reported by other authors (Wang et al. 2015; Samuel et al. 2011; Massányi et al. 1997). It was proved that Cd administration leads to degeneration of corpus lutea, damage and less numerous oocytes, and degeneration of granulosa cells. The rarefaction of granular layer of the follicles observed after Cd administration may lead to disturbances in sex hormones secretion.

## Conclusion

In conclusion, oral exposure of rats to Cd induced damage of ovaries and disturbances of plasma sex hormones levels, which most likely generated morphometric changes in the endometrium and/or abnormalities of sexual cycle phases. Probably for the first time the prolonged effect of Cd action visible as disturbances in estrous cycle, with simultaneous increase in P in plasma, was demonstrated, despite no alteration in concentration of sex hormones in uterine tissue. It suggests that the response of uterus to effects induced by Cd and by 17β-estradiol (positive control) varies both in the morphological study of the endometrium, and in concentration of E_2_ in the uterine tissue, which indicates a different mechanism of Cd action and 17β-estradiol. Thus, this study helps in better understanding of oral Cd toxicity and highlights the risk of the development of reproductive abnormalities, among others menstrual disorders or earlier menopause observed in females exposed to Cd.
